# Anxiety, Depression, and Caregiver Burden Among Family Caregivers of Cancer Survivors

**DOI:** 10.1002/brb3.71258

**Published:** 2026-02-24

**Authors:** Mohammad Al Qadire, Hanan Abdelrahman, Alaa Alanazi, Rama Al Qadire, Omar Al Omari

**Affiliations:** ^1^ Adult Health Department, Faculty of Nursing Al al‐Bayt University Mafraq Jordan; ^2^ College of Nursing and Health Sciences University of Massachusetts Boston Boston Massachusetts USA; ^3^ Faculty of Nursing Suez Canal University Ismailia Egypt; ^4^ College of Nursing Princess Nourah Bint Abdulrahman University Riyadh Saudi Arabia; ^5^ Medical Laboratory, Faculty of Applied Medical Sciences Al al‐Bayt University Mafraq Jordan; ^6^ College of Nursing Sultan Qaboos University Muscat Oman

**Keywords:** anxiety, burden, depression, family caregivers, neoplasm, survivors

## Abstract

**Introduction:**

Family caregivers of cancer survivors face significant psychological challenges, yet their experiences are less studied compared to caregivers of patients undergoing treatment. Therefore, the aim of this study was to assess levels of anxiety, depression, and caregiver burden among family caregivers of cancer survivors and to examine the factors associated with these psychological outcomes.

**Materials and Methods:**

A cross‐sectional survey was conducted among 324 family caregivers recruited from three oncology clinics in Oman.

**Results:**

The mean age of participants was 39.0 years (SD = 11.5), with most being male (62.7%) and employed (54.3%). The prevalence of anxiety and depression among caregivers was 28.4% and 26.2%, respectively. Higher caregiving burden scores were strongly associated with increased odds of anxiety (OR = 1.06, *p* = 0.002) and depression (OR = 1.04, *p* = 0.047). Male caregivers and rural residents reported higher anxiety levels, while unmarried caregivers were at greater risk for depression.

**Conclusions:**

Family caregivers of cancer survivors experience significant psychological distress, particularly those with higher caregiving burden or vulnerable sociodemographic characteristics. These findings underscore the need for targeted mental health interventions such as psychoeducation, emotional support, and access to mental health services to reduce caregiver distress. Addressing their needs is critical to sustaining effective caregiving roles.

## Graphical abstract

In this cross‐sectional study of 324 family caregivers of cancer survivors, 28.4% experienced anxiety and 26.2% experienced depression. Most caregivers (91%) reported mild‐to‐moderate burden (mean = 30.5 ± 6.8). Higher burden, male gender, unmarried status, and rural residence predicted greater psychological distress, highlighting the need for integrated mental health and caregiver support interventions within survivorship care programs.

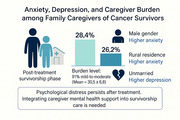

## 1 Background

Cancer is one of the most prevalent and life‐threatening diseases worldwide, significantly affecting the lives of patients and their family caregivers. With advances in cancer treatment, the number of cancer survivors has steadily increased (Karimi Moghaddam et al. 2023). However, the journey of survivorship extends beyond the patient to include family caregivers who provide essential physical, emotional, and psychological support throughout the course of illness (Karimi Moghaddam et al. 2023). These caregivers often experience significant strain, particularly as they assume responsibility for medical tasks, emotional reassurance, financial support, and daily caregiving. The complexity and unpredictability of cancer care place caregivers at risk for a range of adverse mental health outcomes, notably anxiety and depression (O'Rourke et al. 2021; Pan and Lin 2022; Üzar‐Özçeti̇n and Dursun 2020).

The prevalence rates of anxiety and depression among caregivers have varied across studies. Some meta‐analyses report rates as high as 42.3% for depression and 38.1% for anxiety (Pan and Lin 2022; Üzar‐Özçeti̇n and Dursun 2020). In Korea, 38.1% of caregivers experienced anxiety, while 82.2% reported depression. Factors such as caregiving burden, financial strain, and quality of life were linked to these outcomes (Park et al., 2013). Similarly, in Turkey, the prevalence of anxiety and depression was 46.55% and 42.3%, respectively, with caregiving duration and patient functional status playing significant roles (Unsar et al. 2021). However, the only study we identified that specifically focused on family caregivers of cancer survivors was a cross‐sectional survey of 188 caregiver–patient dyads in Germany (Sklenarova et al. 2015). This study revealed that caregivers experienced significantly higher levels of distress, anxiety, and depression than patients they supported. Specifically, 34.9% of caregivers screened positive for anxiety and 26.5% for depression, compared with 26.3% and 28.4%, respectively, among patients (Sklenarova et al. 2015).

Understanding the predictors of anxiety and depression is critical for developing targeted interventions and support. Caregivers’ mental health is influenced by sociodemographic variables, caregiver characteristics, and patient‐related factors (Keramatikerman 2020; Üzar‐Özçeti̇n and Dursun 2020). Younger caregivers, spousal caregivers, and those with limited social support are at a higher risk of anxiety and depression. Additionally, caregivers of patients with advanced‐stage cancer or severe symptoms are more likely to experience heightened emotional distress (Pan and Lin 2022; Üzar‐Özçeti̇n and Dursun 2020). Socioeconomic factors, such as financial strain and job disruption due to caregiving duties, further exacerbate the psychological burden (Alsirafy et al. 2021; Hastert et al. 2020).

Caregiver burden is a multidimensional construct that reflects the physical, emotional, social, and financial hardships experienced by caregivers. Factors such as cancer stage, the patient's level of dependence, and the availability of support systems shape the extent of the caregiver burden. Studies have found a strong correlation between caregiver burden and poor mental health outcomes, particularly anxiety and depression (Alsirafy et al. 2021; Keramatikerman 2020; Üzar‐Özçeti̇n and Dursun 2020). As the burden increases, so does the likelihood of caregivers experiencing psychological distress. The rising number of cancer survivors has placed growing demands on family caregivers who play a vital role in supporting patients throughout the cancer journey. The physical, emotional, and financial demands of caregiving increase their susceptibility to anxiety and depression.

In the present study, the term cancer survivor is used in a more specific sense. While the broader survivorship literature defines cancer survivors as individuals from the time of diagnosis through the remainder of life, including those undergoing active treatment (National Cancer Institute 2024), our study focuses on a subgroup of individuals who have completed active anticancer treatment. These individuals typically attend follow‐up clinics for ongoing surveillance and the management of long‐term or late effects of treatment. This distinction is important because most studies focus on caregivers of current cancer patients, survivorship brings new responsibilities, including managing late effects of treatment, monitoring for recurrence, and supporting emotional adjustment to life after cancer. Studies have shown that caregivers of cancer survivors often experience persistent uncertainty, ongoing vigilance, and sustained caregiving responsibilities, even after active treatment has ended, which can contribute to continued psychological distress (Cui et al. 2025; Kent et al. 2016). However, compared with caregivers of patients in active treatment, caregivers in the survivorship phase receive substantially less clinical attention and fewer structured support services, despite ongoing emotional and practical needs.

In Oman, family caregiving is shaped by cultural expectations that place substantial responsibility on close family members to support individuals living with chronic illness. Care tasks are often shared within the household. Women typically provide routine daily assistance and help with household needs, while men frequently assume responsibilities related to healthcare coordination, such as arranging transportation, accompanying patients to appointments, and communicating with healthcare providers. These patterns reflect broader gender role norms observed in the region. The structure of the healthcare system also influences caregiving demands. Cancer services in Oman are centralized in a small number of tertiary centers located in Muscat, requiring survivors from all regions—including rural and remote areas—to travel for follow‐up care. The experiences of caregivers of cancer survivors are often overlooked. Consequently, there is a critical need for research that addresses the unique experiences and mental health needs of the family caregivers of cancer survivors. To address this gap, this study assessed levels of anxiety, depression, and caregiver burden among family caregivers of cancer survivors and examined the factors associated with these psychological outcomes.

## 2 Methods

### 2.1 Design

This study employed a cross‐sectional, descriptive correlational survey.

### 2.2 Settings

This study was conducted in three outpatient oncology clinics in Oman. The first was Sultan Qaboos University Hospital, a university‐affiliated hospital that primarily treated patients with hematological tumors. The second was the Royal Hospital, a large governmental institution. The third was the Sultan Qaboos Comprehensive Cancer Care and Research Centre, a specialized facility that provided comprehensive treatment for both solid and hematological tumors, as well as conducted cancer research. All three facilities were in Muscat, where most cancer patients in the country received treatment. These settings were well‐suited for recruiting family caregivers providing ongoing support to cancer survivors.

### 2.3 Sample

The target population for this study was the family caregivers of cancer survivors who had completed active cancer treatment and were currently in the survivorship phase. The participants were recruited using purposive sampling from the selected settings. The inclusion criteria for participants included being a family caregiver of a cancer survivor, aged 18 years or older, having the ability to read and understand Arabic, and providing informed consent. Caregivers who self‐reported an existing diagnosed psychiatric disorder or who were currently receiving psychiatric treatment were excluded to avoid confounding pre‐existing mental health conditions with caregiving‐related psychological outcomes.

The estimated sample size was calculated using the Peduzzi et al. (1996) equation (i.e., *N* ≥ 10 *m*) to ensure accurate estimation of model parameters (Peduzzi et al. 1996). Where *N* represents the sample size, and *m* denotes the number of predictors. Given the inclusion of 15 independent variables, a minimum sample size of 150 participants per category of the outcome variable was required. A total of 324 participants were successfully recruited, which was deemed adequate for the statistical requirements of the study.

### 2.4 Data Collection Measures

Data were collected using a structured, self‐administered survey that included the following instruments:

### 2.5 Sociodemographic Questionnaire

Caregivers’ demographic information was collected using a standardized tool. This included data on age, sex, education level, employment status, relationship with the cancer survivor, family history of cancer, presence of other chronic diseases, place of residence, and whether the caregiver lived in the same household as the cancer survivor. Caregivers were also asked a single yes/no question regarding whether they believed they had sufficient cancer‐related knowledge to understand the survivor's diagnosis and follow‐up needs; this item reflected self‐perceived knowledge rather than an objective assessment.

### 2.6 Zarit Burden Interview (ZBI)

The Zarit Burden Interview (ZBI) is a 22‐item scale designed to assess the impact of a patient's disability on a caregiver's emotional, social, physical, and financial well‐being (Zarit et al. 1980). For the first 21 items, respondents rated how often they experienced specific feelings while providing care using a 5‐point Likert scale: never (0), rarely (1), sometimes (2), frequently (3), and nearly always (4). The total ZBI score was obtained by summing the responses across all 22 items, resulting in a possible score range of 0 to 88. Higher scores indicate greater perceived caregiver burden. The total score was categorized as follows: 0–21, no to mild burden; 22–40, mild to moderate burden; 41–60, moderate to severe burden; and ≥ 61, severe burden.

The reliability of the ZBI has been well established, with Cronbach's alpha values consistently reported between 0.85 and 0.92, indicating excellent internal consistency (Bédard et al. 2001). Test‐retest reliability has also demonstrated strong stability, with correlations ranging from 0.71 to 0.85 over varying time intervals (Bédard et al. 2001). In terms of validity, the ZBI has shown strong construct validity, correlating significantly with measures of caregiver stress, depression, and psychological well‐being (Schreiner et al. 2006). Its concurrent validity has been demonstrated through significant associations with other caregiver burden scales, such as the Caregiver Strain Index (Hébert et al. 2000).

### 2.7 Hospital Anxiety and Depression Scale (HADS)

The Hospital Anxiety and Depression Scale (HADS) was developed to measure patient anxiety and depression during hospitalization (Pérez‐San‐Gregorio et al. 2007). It comprises 14 items, with seven items assessing anxiety and seven assessing depression. Patients were required to rate their responses on a scale of 0–3 for each item. The total score for each subscale ranges from 0 to 21; a score of 0 to 7 is considered normal, 8 to 10 borderline abnormal, and 11 to 21 abnormal. For the purpose of logistic regression analysis, we dichotomized the subscale scores into normal (0–7) and clinically relevant symptoms (8–21). Thus, in this study, anxiety refers to a HADS‐A score ≥ 8, and depression refers to a HADS‐D score ≥ 8.

The HADS is a reliable tool, with Cronbach's alpha values ranging between 0.80 and 0.90, indicating good internal consistency. It has been widely used in previous studies conducted in diverse settings (Terkawi et al., 2017). The tool has been validated in Arabic and has strong psychometric properties. In the Arabic version, Cronbach's alpha for the anxiety subscale was 0.83 (95% confidence interval [CI]: 0.79–0.88), and for the depression subscale, it was 0.77 (95% confidence interval: 0.70–0.83) (Terkawi et al., 2017).

### 2.8 Data Collection Procedure

Data were collected over a 12‐week period from April to July 2024. Participants completed the survey using paper‐based forms. The purpose and requirements of the study were explained to the clinic managers to facilitate the data collection process. Research assistants approached potential participants, explained the study to them, and remained on‐site to address any questions and ensure that the surveys were completed accurately.

### 2.9 Ethical Considerations

Ethical approval was obtained from the Institutional Review Board (IRB) of the participating healthcare institutions prior to data collection. All participants received a detailed explanation of the study purpose, procedures, risks, and benefits. Written informed consent was obtained from all participants prior to participation. Participants were informed that participation was voluntary and that they could withdraw from the study at any time without penalty. Confidentiality and anonymity were maintained throughout the study. Participants were assigned unique identification numbers, and no personally identifiable information was linked to their responses. Data were stored in password‐protected electronic files and locked cabinets for the paper‐based surveys. Only the principal investigator and authorized research team members had access to the data.

### 2.10 Data Analysis

All analyses were performed using SPSS (version 29.0; IBM Corp., Armonk, NY, USA). Descriptive statistics were used to summarize demographic information, anxiety, depression, and caregiver burden. Mean, standard deviation, and frequency distributions were calculated for continuous and categorical variables, respectively. Multiple logistic regression analysis was conducted to identify predictors of anxiety and depression. In the logistic regression analysis, we adopted the Hosmer–Lemeshow approach, which involves a two‐step process for fitting multiple logistic regression models. First, simple logistic regression was performed for each independent variable individually. In the second step, a multiple logistic regression model was fitted, incorporating variables that exhibited statistical significance at a pre‐determined conservative threshold (*p* ≤ 0.25). This approach allows for the identification of variables that may not be significant individually but may have combined importance. Statistical significance was set at *p* < 0.05.

## 3 Results

### 3.1 Sample Characteristics

Table 1 presents the demographic characteristics. The mean age of the participants was 39.0 (SD = 11.5) years. Most of the participants were male (62.7%, *n* = 203). Half of the participants (50.3%, *n* = 163) had secondary education, more than half of the participants were employed (54.3%, *n* = 176), and the majority resided in urban areas (58.0%, *n* = 188). Most participants reported no history of chronic disease (86.1%, *n* = 279). A notable proportion of participants (67.3%, *n* = 218) lived in the same household. Most of the participants (78.4%, *n* = 254) demonstrated cancer‐related knowledge. Regarding marital status, 74.4% (*n* = 241) of participants were married. The care providers were primarily first‐degree relatives (74.1%; *n* = 240). More specifically, caregivers represented a range of close family roles: spouses constituted the largest group (40.4%, *n* = 115), followed by parents (20.7%, *n* = 59), siblings (15.1%, *n* = 43), sons or daughters (8.1%, *n* = 23), and other relatives (8.4%, *n* = 24).

**TABLE 1:** Participants demographics characteristics.

Variable		Frequency (%)	Mean (SD)
Age (year)			39.0 (11.5)
Sex	Female	121 (37.3)	
	Male	203 (62.7)	
Employment status	Unemployed	148 (45.7)	
	Employed	176 (54.3)	
Place of living	Rural	136 (42.0)	
	Urban	188 (58.0)	
Chronic disease	No	279 (86.1)	
	Yes	45 (13.9)	
Same household	No	106 (32.7)	
	Yes	218 (67.3)	
Family cancer history	No	270 (83.3)	
	Yes	54 (16.7)	
Cancer knowledge	No	70 (21.6)	
	Yes	254 (78.4)	
Education level	Secondary and less	163 (50.3)	
	Diploma and above	161 (49.7)	
Marital status	Not married	83 (25.6)	
	Married	241 (74.4)	
Care provider	First degree relative	240 (74.1)	
	Others	84 (25.9)	

### 3.2 Anxiety, Depression, and Caregiver Burden

Anxiety assessment yielded a mean total score of 5.3 (SD =  4.4), with values ranging from 0 to 20. A significant proportion of the cohort (71.6%, *n* = 232) exhibited normal levels, 13.6% (*n* = 44) were classified as borderline abnormal, and 14.8% (*n* = 48) were classified as abnormal. For the depression subscale, the mean total score was 4.6 (SD = 3.9), ranging from 0 to 20. Most participants (73.8%, *n* = 239) were within the normal range, with 17.6% (*n* = 57) categorized as borderline abnormal and 8.6% (*n* = 28) as abnormal (Table 2). The burden evaluation yielded a mean total score of 30.5 (SD = 6.8), encompassing a range of 22–56. An overwhelming majority of the sample (91.0%, *n* = 295) reported experiencing a mild‐to‐moderate burden, whereas 9.0% (*n* = 29) reported moderate‐to‐severe burden levels.

**TABLE 2:** Anxiety and depression scores among family caregivers of cancer survivors (*N* = 324).

Category	Anxiety *n* (%)	Depression *n* (%)
Normal (0–7)	232 (71.6%)	239 (73.8%)
Borderline (8–10)	44 (13.6%)	57 (17.6%)
Abnormal (11–21)	48 (14.8%)	28 (8.6%)

### 3.3 Predictors of Anxiety and Depression

Using the Hosmer–Lemeshow approach, which involves a two‐step process for fitting multiple logistic regression models to identify predictors of anxiety and depression, the first step identified the following variables for inclusion in the multiple logistic regression model for anxiety: age (*p* = 0.146), sex (*p* = 0.011), marital status (*p* = 0.040), place of residence (*p* = 0.119), family cancer history (*p* = 0.025), cancer knowledge (*p* = 0.009), care provider (*p* = 0.029), and total burden score (*p* = 0.010) (Table 3). For depression, the first step identified the following variables for inclusion in the multiple logistic regression model: sex (*p* = 0.063), marital status (*p* = 0.153), work status (*p* = 0.137), place of residence (*p* = 0.055), education level (*p* = 0.016), and total burden score (*p* = 0.027), (see Table 4).

**TABLE 3:** Simple logistic regression analysis of the association between demographic variables and anxiety status.

Variable	B	df	*p* Value	Odds ratio	95% CI
**Age**	−0.016	1	0.146	0.98	0.96 to 1.01
**Sex**	0.635	1	0.011	1.89	1.16 to 3.07
**Marital status**	−0.424	1	0.040	0.66	0.44 ‐ 0.98
**Work status**	0.215	1	0.328	1.24	0.80 to 1.91
**Place of living**	0.312	1	0.119	1.37	0.92 to 2.03
**Chronic disease**	−0.189	1	0.415	0.83	0.54 to 1.27
**Same household**	−0.109	1	0.564	0.90	0.62 to 1.28
**Family cancer history**	−0.543	1	0.025	0.58	0.36 to 0.93
**Cancer knowledge**	0.785	1	0.009	2.19	1.21 to 3.96
**Education level**	0.016	1	0.943	1.02	0.64 to 1.60
**Care provider**	0.601	1	0.029	1.82	1.06 to 3.13
**Total burden score**	0.045	1	0.010	1.05	1.01 to 1.08

**TABLE 4:** Simple logistic regression analysis of the association between demographic variables and depression status.

Variable	B	df	*p* Value	Odds ratio	95% CI
**Age**	0.004	1	0.710	0.99	0.97 to 1.01
**Sex**	−0.427	1	0.063	0.652	0.41 to1.03
**Marital Status**	0.256	1	0.153	1.291	0.90 to 1.84
**Work Status**	−0.315	1	0.137	0.73	0.48 to 1.09
**Place of Living**	0.409	1	0.055	1.505	0.99 to 2.27
**Chronic Disease**	−0.218	1	0.351	0.804	0.51 to 1.25
**Same Household**	0.021	1	0.911	1.021	0.70 to 1.47
**Family Cancer History**	−0.186	1	0.462	0.83	0.51 to 1.34
**Cancer Knowledge**	−0.401	1	0.158	0.67	0.39 to 1.15
**Education Level**	−0.546	1	0.016	0.579	0.38 to 0.88
**Care Provider**	0.113	1	0.668	1.12	0.66 to 1.89
**Total Burden Score**	0.04	1	0.027	1.041	1.01 to 1.07

These variables were used to construct two logistic regression models. The results of the multiple logistic regression analysis of predictors of anxiety are presented in Table 5. This analysis identified several significant predictors of anxiety. Sex was a significant predictor, with females having lower odds of anxiety than males (B = −0.59, *p* = 0.046, OR = 0.55, 95% CI: 0.31, 0.99). Place of living was also a significant predictor, with individuals living in urban areas having lower odds of anxiety than those living in rural areas (B = −0.71, *p* = 0.011, OR = 0.49, 95% CI: 0.29, 0.85). The total burden score was significantly associated with higher odds of anxiety (B = 0.062, *p* = 0.002, OR = 1.06, 95% CI: 1.02, 1.11). Marital status (B = −0.45, *p* = 0.164, OR = 0.64, 95% CI: 0.34 to 1.20), family cancer history (B = 0.08, *p* = 0.812, OR = 1.09, 95% CI: 0.53 to 2.22), cancer knowledge (B = 0.48, *p* = 0.184, OR = 1.62, 95% CI: 0.79 to 3.31), and care provider status (B = −0.08, *p* = 0.812, OR = 0.92, 95% CI: 0.44 to 1.91) were not significantly associated with anxiety in the multiple logistic regression model. The Cox & Snell R‐Square (0.190) and Nagelkerke R‐Square (0.229) indicate that the model explains approximately 19% to 22.9% of the variance in the outcome variable.

**TABLE 5:** Multiple logistic regression analysis of predictors of anxiety.

Predictor	B	df	*p* Value	Odds ratio	95% CI
**Sex** (female with reference to male)	−0.59	1	0.046	0.55	0.31 to 0.99
**Marital status** (married with reference to not married)	−0.45	1	0.164	0.64	0.34 to 1.20
**Place of living** (urban with reference to rural)	−0.71	1	0.011	0.49	0.29 to 0.85
**Family cancer** h**istory** (yes with reference to no)	0.08	1	0.812	1.09	0.53 to 2.22
**Cancer knowledge** (yes with reference to no)	0.48	1	0.184	1.62	0.79 to 3.31
**Care provider** (first degree relative with reference to others)	−0.08	1	0.812	0.92	0.44 to 1.91
**Total burden score**	0.062	1	0.002	1.06	1.02 to 1.11
Constant	−2.066	1	0.008	0.13	

The results of the multiple logistic regression analysis for the predictors of depression are presented in Table 6. The analysis identified the significant predictors of depression. Marital status was a significant predictor, with married participants having lower odds of depression than unmarried participants (B = −0.61, *p* = 0.035, OR = 0.54, 95% CI: 0.31 0.96). The total burden score was also significantly associated with higher odds of depression (B = 0.04, *p* = 0.047, OR = 1.04, 95% CI: 1.0 to 1.08). Education level (B = −0.26, *p* = 0.311, OR = 0.77, 95% CI: 0.46 to 1.28) and sex (B = −0.16, *p* = 0.571, OR = 0.86, 95% CI: 0.50 to 1.47) were not significantly associated with depression in the multiple logistic regression model. The Cox & Snell R‐Square (0.134) and Nagelkerke R‐Square (0.150) indicate that the model explains approximately 13.4% to 15.0% of the variance in the outcome variable.

**TABLE 6:** Multiple logistic regression analysis of predictors of depression.

Predictor	B	df	*p* Value	Odds ratio	95% CI
**Marital status** (married with reference to not married)	−0.61	1	0.035	0.54	0.31 to 0.96
**Education level** (diploma and above, with reference to secondary and less)	−0.26	1	0.311	0.77	0.46 to 1.28
**Total burden score**	0.04	1	0.047	1.04	1.0 to 1.08
**Sex** (female with reference to male)	−0.16	1	0.571	0.86	0.50 to 1.47
**Constant**	−1.49	1	0.021	0.23	

## 4 Discussion

### 4.1 Prevalence of Anxiety and Depression Among Caregivers of Cancer Survivors

Our study highlights the prevalence of anxiety and depression among family caregivers of survivors of cancer. A systematic review aimed at assessing the prevalence of anxiety and depression among older adult cancer survivors identified only two studies on anxiety and four on depression, all of which were conducted before 2015. This underscores the scarcity of evidence addressing these mental health issues among the family caregivers of cancer survivors. The findings of our study are consistent with those of other similar studies. For instance, anxiety rates ranging from 25% to 38.6% and depression rates between 22.7% and 30% have been reported in previous studies (Jansen et al. 2018) Similarly, while another study investigating the psychological well‐being and support needs of informal caregivers of cancer survivors revealed that 34.9% of caregivers experienced anxiety and 26.5% experienced depression, highlighting the significant emotional burden faced by caregivers (Sklenarova et al. 2015).

Symptoms of anxiety and depression among family caregivers of cancer survivors were comparable to those experienced by caregivers of cancer patients undergoing active treatment. For instance, a systematic review reported prevalence rates of 46.55% for anxiety and 42.30% for depression among caregivers of patients currently receiving treatment (Pan and Lin 2022). However, the relatively lower rates of anxiety observed in our study may be attributed to differences in caregiving stages. Our study focuses on caregivers of cancer survivors who are likely to experience a different set of stressors than those supporting patients undergoing active treatment.

Caregivers of cancer survivors may experience less acute stress because the immediate life‐threatening nature of the disease has passed, and survivors may require fewer medical interventions and caregiving tasks than those in active treatment phases. This reduction in caregiving intensity can lead to a sense of emotional relief and improved psychological well‐being. Additionally, witnessing the improved prognosis of cancer survivors and their gradual return to daily life may provide caregivers with a sense of hope and accomplishment, further mitigating stress and anxiety.

However, it is essential to consider that caregiving for cancer survivors comes with unique challenges, such as the fear of cancer recurrence, which may contribute to lingering anxiety. Recurrence can create a persistent sense of uncertainty and worry even when survivors appear to be in remission (O'Rourke et al., 2021). Moreover, caregivers often navigate long‐term responsibilities, such as managing survivors’ follow‐up appointments, handling financial burdens, and addressing survivors’ lingering physical or emotional side effects from treatment, all of which can contribute to psychological strain (Hastert et al., 2020). These findings suggest that the caregiving stage plays a critical role in shaping caregivers' emotional experiences. While caregivers of survivors may face less immediate medical and caregiving demands, the enduring nature of psychological stressors, such as uncertainty and financial concerns, emphasizes the need for targeted mental health support interventions that address their specific challenges. For example, brief, structured caregiver support programs focusing on stress management, role negotiation within families, and problem‐solving strategies may be beneficial.

### 4.2 Burden of Family Caregivers of Cancer Survivors

The current study revealed a mean burden score of 30.5 (SD = 6.8), with 91.0% of family caregivers experiencing a mild‐to‐moderate burden. These results are consistent with the general trend observed in previous studies on caregiver burden, which commonly highlights mild‐to‐moderate burden as the most prevalent level among caregivers of patients with cancer (Alsirafy et al. 2021; Karimi Moghaddam et al. 2023; Kondeti et al. 2021; Onyeneho and Ilesanmi 2021). Across multiple contexts, family caregivers frequently report moderate levels of burden, with cultural, healthcare system, and socioeconomic factors influencing the extent of the burden. For example, a study among caregivers of patients with cancer in Nigeria reported that the majority of caregivers experienced a mild‐to‐moderate burden. This suggests that, regardless of regional or cultural differences, caregivers experience comparable burden levels, likely due to the physical, emotional, and psychological demands of caregiving.

In contrast to our findings, significantly higher levels of burden were reported among spousal family caregivers (M = 45.76, SD = 14.66) (Tao et al. 2022), caregivers of patients undergoing chemotherapy (M = 38.98, SD = 10.53) (Mishra et al. 2021), and informal caregivers of children (M = 68.4, SD = 1.5) (Chaghazardi et al. 2022). These differences underscore the influence of culture, healthcare infrastructure, contextual factors, and caregiver role expectations on shaping caregiver burden trends across various regions. Moreover, support systems, access to healthcare, and intervention programs play pivotal roles in how caregivers perceive and manage their burden. This emphasizes the critical need for tailored support strategies, such as psychoeducation and caregiver skill training, to mitigate the burden and prevent its escalation from moderate to severe levels.

### 4.3 Predictors of Anxiety and Depression

#### 4.3.1 Anxiety

In this study, sex emerged as a significant predictor of anxiety, with male caregivers experiencing higher odds of anxiety than their female counterparts did. This finding aligns with prior research suggesting that men, who are often unaccustomed to caregiving roles, may struggle more with emotional regulation and may have limited access to informal support networks compared to women (Karimi Moghaddam et al. 2023; Park et al. 2013; Sklenarova et al. 2015). Additionally, place of residence significantly influenced anxiety levels, with caregivers living in urban areas reporting lower anxiety than those living in rural areas. Rural caregivers face barriers to healthcare access, reduced availability of support services, and isolation, all of which exacerbate their anxiety (Alsirafy et al. 2021; Hastert et al. 2020). The availability of support systems and mental health services in urban areas may explain the lower anxiety levels observed among urban caregivers. Caregiving burden was also a strong predictor of anxiety. The findings indicated that higher caregiving burden scores were significantly associated with increased odds of anxiety, consistent with the findings of previous meta‐analyses (García‐Torres et al. 2020; Karimi Moghaddam et al. 2023; Keramatikerman 2020; Üzar‐Özçeti̇n and Dursun 2020). Caregivers tasked with the physical, emotional, and financial responsibilities of caregiving face cumulative stress, leading to anxiety. The unpredictability of disease progression and the constant need to adapt to new caregiving demands can exacerbate feelings of helplessness.

#### 4.3.2 Depression

The findings indicated that marital status was a significant predictor of depression, with unmarried caregivers showing higher odds of depression than their married counterparts. This finding reflects the protective effect of spousal support, as married caregivers have greater emotional and practical support to help manage the challenges of caregiving (García‐Torres et al. 2020; Karimi Moghaddam et al. 2023; Pan and Lin 2022). The presence of a spouse offers opportunities for shared responsibility and emotional reassurance, thus mitigating the impact of caregiving stress. Caregiving burden has emerged as a significant predictor of depression, with higher burden scores linked to an increased likelihood of depression. This association has been well‐documented in the literature, as caregiving burden represents the cumulative impact of physical, emotional, social, and financial strain on caregivers (Keramatikerman 2020; Kondeti et al. 2021; Yu et al. 2021). In the context of cancer survival, burden is compounded by the long‐term nature of care. Caregivers must continue to provide support as survivors navigate the post‐treatment rehabilitation, financial strain, and potential late effects of cancer treatment. Over time, the persistence of these challenges contributes to emotional exhaustion and depressive symptoms.

These findings underscore the critical need for mental health interventions specifically tailored to the caregivers of cancer survivors. Healthcare providers should prioritize the implementation of targeted strategies to alleviate caregiver burden. Interventions such as respite care programs, caregiver education, and mental health support services can play a pivotal role in reducing anxiety and depression among family caregivers of survivors of cancer.

#### 4.3.3 Limitations

This study has several limitations. First, the cross‐sectional design limited the ability to establish causal relationships between caregiving burden, anxiety, and depression. Second, the use of self‐reported measures may introduce response bias, as participants could underreport or overreport their mental health symptoms. Third, although the study provides insight into the psychological well‐being of caregivers of cancer survivors, we did not collect detailed survivor‐related characteristics such as age, cancer type, treatment modality, disease stage, or time since diagnosis. These factors may influence caregiving responsibilities and could potentially help in understanding variations in caregiver anxiety, depression, and burden. The absence of survivor clinical data may therefore limit the extent to which we can explore possible relationships between survivor characteristics and caregiver outcomes or compare findings across different clinical contexts. Fourth, caregivers were recruited from those accompanying survivors to clinic visits, which may not represent all individuals involved in home‐based caregiving. Although these accompanying caregivers may also participate in some home care tasks, this was not verified in our study. Finally, fear of recurrence has been widely recognized as a significant source of anxiety for caregivers, with research indicating that caregivers often experience higher levels of anxiety than survivors (O'Rourke et al. 2021). However, this study did not examine fear of recurrence or its influence on anxiety and depression, which may account for the limited percentage of variance explained by the regression models.

### 4.4 Implications for Practice

The findings suggest the need to integrate caregiver support into routine survivorship follow‐up rather than only during active treatment. Nurses in oncology outpatient clinics and primary care settings are often the first point of contact during follow‐up appointments and are in a position to identify caregiver distress through brief screening tools such as HADS or caregiver burden scales. Their role may include providing brief psychoeducation on coping strategies, facilitating referrals to psychosocial support services, or coordinating telehealth support for caregivers who travel long distances. This approach aligns with existing survivorship care workflows and allows for practical, feasible integration of caregiver support without requiring intensive new service structures.

## 5 Conclusion

This study highlights anxiety and depression as critical mental health challenges faced by the family caregivers of cancer survivors. Caregiving burden and sociodemographic factors such as sex, marital status, and place of residence were identified as key predictors of psychological outcomes. Despite the transition to survivorship, family caregivers continue to exhibit notable levels of anxiety and depression, underscoring the importance of integrating targeted and accessible psychological support (e.g., psychoeducation, emotional support) within survivorship care pathways. Future research should prioritize longitudinal studies to better understand the evolving nature of caregivers’ mental health and develop more effective support systems.

## Author Contributions

All authors made substantial contributions to the conception and design of the study, data collection, analysis, and manuscript preparation. **Mohammad Al Qadire**: conceptualized the study, secured funding, supervised the research process, and contributed to methodology development, drafting, and critical revision of the manuscript. **Hanan Abdelrahman**: coordinated data collection, conducted formal analysis, and contributed to manuscript writing and correspondence. **Alaa Alanazi**: provided additional funding support, resources, validation, and participated in manuscript review. **Rama Al Qadire**: assisted with data collection, curation, and visualization, and contributed to manuscript editing. **Omar Al Omari**: contributed to study methodology, statistical analysis, supervision, and manuscript review. All authors reviewed and approved the final version of the manuscript and agree to be accountable for all aspects of the work.

## Funding

This work was supported by the Ministry of Higher Education, Research and Innovation, Oman, with reference number (RC/RG‐CON/AHCC/22/01) and Princess Nourah Bint Abdulrahman University Researchers Supporting Project number (PNURSP2025R717), Princess Nourah Bint Abdulrahman University, Riyadh, Saudi Arabia.

## Ethics Statement

This study was performed in line with the principles of the Declaration of Helsinki. Approval was granted by the Ethics Committee of Sultan Qaboos University Hospital (REF. NO. SQU‐EC/ 286/2022), Ministry of Health (MoH/CSR/22/25408), and Sultan Qaboos Comprehensive Cancer Care and Research Centre (SQCCC&RC) (CCCRC‐08‐2022).

## Consent

Informed consent was obtained from all individual participants included in the study.

## Conflicts of Interest

The authors declare no conflicts of interest.

## Data Availability

The datasets generated during and/or analyzed during the current study are available from the corresponding author on reasonable request.
